# Porous two dimensional architectures for high efficiency electrocatalytic urea oxidation

**DOI:** 10.1002/smo2.70057

**Published:** 2026-05-06

**Authors:** Kean Zhu, Zebo Li, Ruchuan Chen, Linai Zhou, Yujie Ma, Jun Wan

**Affiliations:** ^1^ State Key Laboratory of New Textile Materials and Advanced Processing School of Chemistry and Chemical Engineering Wuhan Textile University Wuhan Hubei China; ^2^ Department of Chemistry University of Manchester Manchester UK

**Keywords:** 2D material, electronic configuration, porous material, small‐molecule oxidation, urea oxidation

## Abstract

Porous two‐dimensional (2D) materials have emerged as promising electrocatalysts for the urea oxidation reaction (UOR) due to their high surface area, tunable electronic properties, and enhanced charge and mass transport. These structural advantages improve catalytic activity, selectivity, and reaction kinetics. However, challenges such as catalyst deactivation, structural instability, and limited mechanistic understanding still restrict their broader application. While previous studies have explored compositional tuning and catalytic performance, a systematic understanding of how porosity, defect engineering, and electronic structure modulation govern UOR efficiency and durability remains limited. This review comprehensively evaluates porous 2D materials for UOR, focusing on structure–activity relationships and fundamental catalytic mechanisms. Special attention is given to how pore architecture, surface defects, and electronic configurations regulate reaction pathways. Porous 2D materials are categorized into six major classes: oxides and their derivatives, hydroxides, sulfides, phosphides, carbides and nitrides, and other emerging materials. Unlike conventional reviews that primarily classify materials based on composition and performance, this work highlights the role of microstructural design in optimizing UOR electrocatalysis. By addressing existing knowledge gaps and identifying key structural parameters for catalyst optimization, this review provides a systematic framework for guiding the rational design of porous 2D materials in sustainable electrochemical energy conversion.

## Graphical abstract

This review elucidates how porous two‐dimensional architectures regulate urea oxidation pathways through coupled structural and electronic effects, offering structure‐guided insights into catalytic efficiency, stability, and low‐carbon energy conversion.

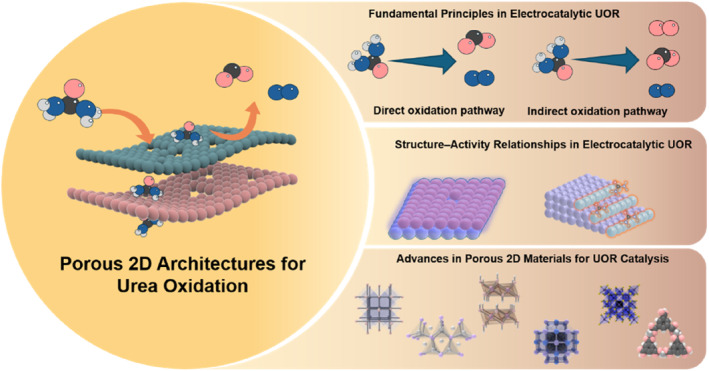

## 1 INTRODUCTION

The urea oxidation reaction (UOR) is a crucial anodic electrocatalytic process widely applied in urea fuel cells and urea‐assisted water electrolysis for hydrogen production.[^1^, ^2^, ^3^] Compared to the conventional oxygen evolution reaction (OER), UOR exhibits a significantly lower thermodynamic onset potential (0.37 V vs. RHE, whereas OER requires 1.23 V vs. RHE),^[4]^ effectively reducing the energy consumption associated with hydrogen production.[^5^, ^6^, ^7^] Moreover, since urea is abundant in industrial wastewater, agricultural runoff, and biological waste, its electrocatalytic oxidation enables both nitrogen pollution mitigation and simultaneous hydrogen recovery, thus integrating environmental remediation with resource utilization.[^8^, ^9^, ^10^] Consequently, UOR has garnered significant attention in the fields of sustainable energy and environmental science, emerging as a focal point of electrocatalysis research.[^11^, ^12^, ^13^] However, due to its six‐electron transfer nature, UOR follows a complex reaction pathway, leading to sluggish kinetics that hinder its practical application.^[14]^ Recent studies highlight that the initial adsorption of urea molecules onto the catalyst surface plays a pivotal role in UOR.^[15]^ However, conventional catalysts often suffer from suboptimal active site distribution, density, and surface affinity, limiting the efficiency of urea activation.^[16]^ Additionally, the cleavage of the C–N bond, a rate‐determining step (RDS) in UOR, is strongly influenced by electron transfer efficiency.^[17]^ Many catalysts lack the precise electronic structure required to regulate the generation and decomposition of reaction intermediates, leading to the accumulation of byproducts (e.g., HNCO) and diminished catalytic selectivity.[^17^, ^18^] Moreover, catalyst deactivation may occur due to the adsorption of reaction intermediates on active sites, while insufficient intrinsic conductivity in some materials further restricts electron transport, thereby impeding overall catalytic kinetics.[^19^, ^20^] Consequently, rational catalyst design aimed at optimizing urea adsorption, modulating electronic structure, enhancing charge transport, and suppressing byproduct formation is essential for improving UOR performance.[^21^, ^22^]

In the pursuit of optimizing UOR catalysts, porous two‐dimensional (2D) materials have emerged as highly promising candidates due to their distinctive structural advantages. Unlike conventional bulk materials, 2D materials exhibit atomic‐scale thickness and tunable interlayer stacking, endowing them with unique electronic structures and short‐range charge transport pathways.^[23]^ The incorporation of porosity further enhances their catalytic efficiency by increasing the surface area, augmenting the density of active sites, and facilitating the diffusion of reactants and intermediates.[^24^, ^25^, ^26^] These synergistic effects render porous 2D materials particularly advantageous for UOR electrocatalysis.^[27]^ A primary advantage of porous structures is their ability to provide a high density of catalytically active sites. Given that the adsorption and activation of urea molecules is a critical step in UOR, an increased surface area enables greater contact with reactant species, thereby improving catalytic efficiency. Moreover, well‐defined pore architectures promote uniform electrolyte infiltration, ensuring a more homogeneous reaction interface and enhanced overall catalytic performance.^[28]^ Recent reviews have further emphasized that mesostructured pore and channel architectures play a crucial role in heterogeneous catalysis by regulating mass transport, reactant accessibility, and intermediate diffusion within catalytic interfaces.^[29]^ In addition to improving mass transport, porous structures also contribute to optimizing electron transfer.[^30^, ^31^] Since UOR is a multi‐electron reaction, sluggish charge transfer kinetics can often limit the catalytic process.^[28]^ By introducing interconnected pore networks within 2D materials, charge transport pathways are shortened, electronic resistance is reduced, and local electronic environments at active centers can be modulated, ultimately enhancing catalytic activity.^[32]^ Despite these advantages, porous 2D materials still face several critical challenges in UOR applications. Recent studies have continued to advance the design of highly efficient electrocatalysts for urea oxidation, highlighting the rapid evolution of this field and the growing importance of rational catalyst engineering.^[33]^ Although experimental studies have demonstrated that porosity can enhance catalytic activity, the underlying mechanisms remain insufficiently elucidated.^[34]^ Current optimization strategies largely rely on empirical tuning rather than a systematic theoretical framework, limiting the precise control of catalytic performance.[^35^, ^36^, ^37^] Additionally, structural instability remains a concern, as certain porous 2D materials may undergo dissolution, pore collapse, or structural reconfiguration under UOR conditions, leading to performance degradation over time.[^38^, ^39^, ^40^] Furthermore, achieving scalable fabrication while preserving catalytic efficiency remains a pressing issue.[^41^, ^42^, ^43^] To address these challenges, future research should focus on establishing a deeper understanding of the structure‐activity relationships governing porous 2D materials, alongside the development of robust and industrially viable catalysts for practical UOR applications.[^44^, ^45^, ^46^]

**FIGURE 1:**
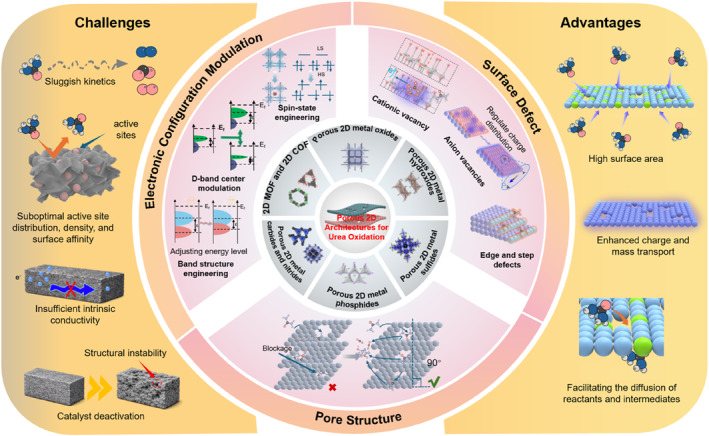
Schematic overview of porous 2D materials for electrocatalytic urea oxidation.

This review systematically investigates the structural characteristics of porous 2D materials and their impact on UOR catalysis. By analyzing recent advances and fundamental mechanisms, this work elucidates how microstructural features influence catalytic activity, charge transfer kinetics, and long‐term stability, providing insights for the rational design of high‐efficiency UOR electrocatalysts. To achieve this, we categorize these materials into six major classes: porous 2D oxides and their derivatives, hydroxides, sulfides, phosphides, carbides and nitrides, and other emerging porous 2D materials (Figure 1). A detailed discussion is provided on their catalytic activity, charge transport properties, and electrochemical stability, with a particular focus on how porosity, surface defects, and electronic structure modulation influence reaction pathways to enhance activity, selectivity, and durability. Unlike conventional reviews that primarily summarize material classifications and catalytic performance, this work emphasizes the intrinsic structure–performance relationship, highlighting the role of molecular‐level structural tuning in optimizing UOR efficiency. Through this analysis, the review provides a comprehensive understanding of porous 2D materials in UOR catalysis and offers a forward‐looking perspective on the development of next‐generation electrocatalysts.

## 2 FUNDAMENTAL PRINCIPLES AND STRUCTURE–ACTIVITY RELATIONSHIPS IN ELECTROCATALYTIC UREA OXIDATION

Electrocatalytic urea oxidation is governed by intricate reaction mechanisms, electronic interactions, and structural dynamics that collectively determine catalytic efficiency.[^47^, ^48^, ^49^] Among the various electrocatalyst designs, porous 2D materials have emerged as highly promising candidates due to their high surface area, tunable electronic properties, and enhanced charge and mass transport.[^50^, ^51^] This section systematically explores the mechanistic principles of UOR, the structural advantages of porous 2D materials, and the structure–activity relationships that dictate the catalytic performance.^[52]^ Specifically, we categorize porous 2D materials into six major classes, including oxides and their derivatives, hydroxides, sulfides, phosphides, carbides and nitrides, and other emerging materials, each exhibiting distinct catalytic characteristics and stability trends in UOR applications.

### 2.1 Mechanism and reaction pathways of urea oxidation

**FIGURE 2:**
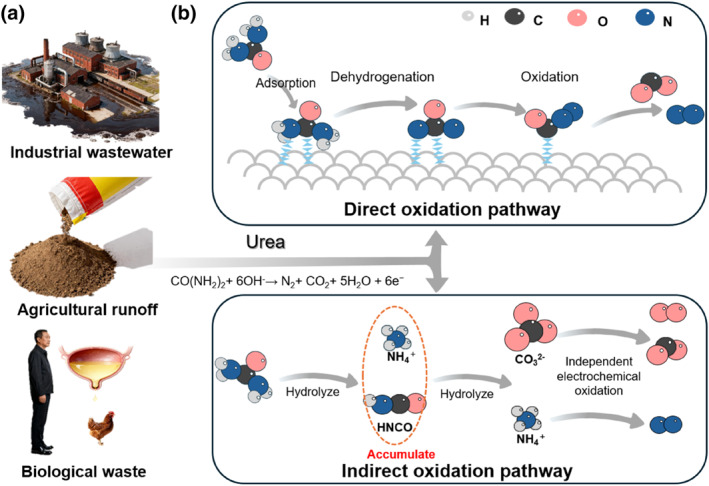
Schematic illustration of urea sources and electrocatalytic oxidation pathways. (a) Major sources of urea in the environment. (b) Direct and indirect oxidation pathways of urea.

Urea, a primary nitrogen‐containing compound in human and animal wastes, industrial effluents, and agricultural runoff, represents a major contributor to water pollution and nitrogen‐cycle imbalance. As illustrated in Figure 2a, diverse sources—including industrial wastewater, excessive fertilizer leaching, and biological excreta—led to widespread urea accumulation in natural water bodies. This not only threatens aquatic ecosystems through eutrophication but also challenges wastewater remediation efforts on a global scale. From an energy perspective, urea is also a high hydrogen‐density molecule, offering a promising low‐energy alternative to oxygen evolution in electrolysis‐driven hydrogen production. Consequently, the UOR serves as a strategic anodic pathway for simultaneous clean energy generation and nitrogen pollutant mitigation, making it highly attractive in the context of decarbonized energy systems and environmental sustainability. The UOR is a complex multi‐electron anodic process, where reaction kinetics are governed by multiple coupled electron‐proton transfer steps.[^53^, ^54^] These challenges impose strict requirements on the catalyst electronic structure, active site distribution, and interfacial charge transport properties.[^55^, ^56^] Thermodynamically, the UOR has a significantly lower theoretical onset potential (0.37 V vs. RHE)^[57]^ compared to the OER (1.23 V vs. RHE), enabling reduced energy consumption for electrolysis.[^15^, ^58^] However, despite its potential in energy conversion and wastewater treatment, UOR remains kinetically sluggish, requiring high overpotentials that hinder its practical efficiency.[^59^, ^60^, ^61^] A fundamental understanding of the reaction mechanism is thus essential for guiding the rational design of highly active catalysts.[^34^, ^62^] The overall UOR process follows the reaction (Figure 2b): ${\text{CO}\left({\text{NH}}_{2}\right)}_{2}+6{\text{OH}}^{-}\to N_{2}+{\text{CO}}_{2}+5H_{2}O+6e^{-}$.^[63]^ This reaction proceeds through several key steps, including initial urea adsorption, C–N bond cleavage, transformation of nitrogen‐containing intermediates, and final product desorption.[^64^, ^65^] Based on reaction pathways, UOR is categorized into two main mechanisms: (1) the direct oxidation pathway, in which urea undergoes sequential dehydrogenation and oxidation at the catalyst surface, ultimately yielding carbon dioxide and nitrogen gas, and (2) the indirect oxidation pathway, where urea first hydrolyzes into carbonate and ammonia, followed by their independent electrochemical oxidation.^[66]^ The direct pathway requires efficient C–N bond cleavage,^[67]^ while the indirect pathway often leads to the accumulation of byproducts such as HNCO and NH_4_^+^, affecting catalytic selectivity.[^22^, ^64^, ^68^] The catalyst's electronic configuration and surface properties thus play a crucial role in determining the preferred reaction route. C–N bond cleavage is widely considered the RDS in UOR due to its high activation energy barrier, necessitating catalyst design strategies that optimize electronic states to facilitate bond breaking.[^15^, ^69^] Both theoretical and experimental studies indicate that oxygen vacancies, metal coordination environments, and local electronic states strongly influence the reaction pathway.[^70^, ^71^, ^72^] For instance, oxygen vacancy‐rich catalysts can modulate surface electronic structures, inducing slight elongation of adsorbed urea's C–N bond, thereby reducing the energy barrier for cleavage.^[73]^ Additionally, the oxidation state of metal centers dictates intermediate adsorption strength, impacting both reaction selectivity and kinetics.^[74]^ The presence of strongly adsorbed intermediates (e.g., HNCO, N_2_H_4_, NH_2_^−^) can lead to active site poisoning, limiting long‐term catalytic stability.[^75^, ^76^] Thus, an ideal catalyst should not only promote efficient C–N bond scission but also optimize adsorption energies to prevent unwanted intermediate accumulation, ensuring enhanced activity and durability.^[77]^

### 2.2 Structural and electronic advantages of porous 2D materials in UOR

**FIGURE 3:**
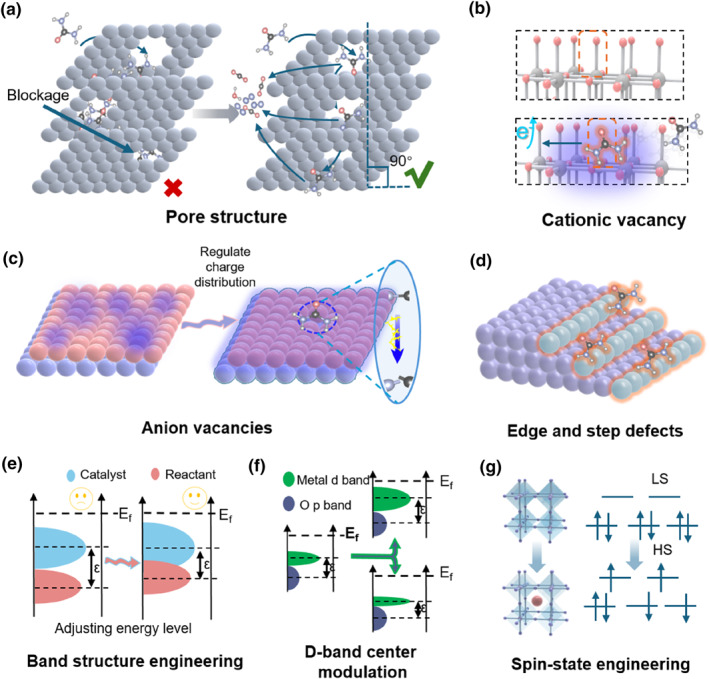
Schematic illustration of structure–activity regulation in porous 2D materials for electrocatalytic urea oxidation. (a) Surface‐congested adsorption impedes reaction kinetics, while porous surface channels enhance molecular accessibility and diffusion. (b) Edge‐rich defect architectures promote adsorption and facilitate dehydrogenation via exposed active sites. (c) Oriented electron–proton transfer pathways in layered structures accelerate charge separation and intermediate conversion. (d) Interlayer confinement and channelized transport enhance molecular activation and reduce energy barriers. (e) Nanopore‐induced confinement selectively stabilizes reaction intermediates and accelerates C–N bond scission. (f) Electronic structure tuning via vacancy or coordination modulation shifts the d‐band center, optimizing adsorption energy. (g) Orbital symmetry manipulation under crystal field splitting enables precise control over catalytic site reactivity.

Porous 2D materials have demonstrated significant potential in enhancing the catalytic efficiency of the UOR due to their unique layered structures, high surface area, and tunable porosity.[^78^, ^79^, ^80^] Unlike bulk or zero‐dimensional nanomaterials, 2D catalysts benefit from atomically thin layers that maximize active site exposure while minimizing electron and ion transport resistance.[^81^, ^82^, ^83^] However, achieving high catalytic performance in UOR requires more than just an increased surface area; the pore architecture, surface defects, and electronic configurations play decisive roles in determining catalytic activity, reaction selectivity, and long‐term stability (Figure 3).[^84^, ^85^, ^86^]

### Pore architecture

Pore architecture is a critical factor in optimizing mass transport and active site accessibility, particularly for diffusion‐limited reactions such as UOR.[^87^, ^88^] Based on pore size, porous structures can be categorized into three main types: (1) micropores (<2 nm), which significantly enhance the surface area and facilitate strong physical adsorption of urea molecules; (2) mesopores (2–50 nm), which improve electrolyte penetration and reduce ion diffusion resistance,^[89]^ thereby enhancing catalytic efficiency[^90^, ^91^]; and (3) macropores (>50 nm), which provide open pathways for rapid reactant transport, minimizing mass transfer limitations and ensuring homogeneous reaction kinetics.[^92^, ^93^] In UOR, the diffusion, adsorption, and oxidation of urea involve multiple reaction interfaces, making interconnected pore networks essential for efficient catalytic performance.[^94^, ^95^, ^96^] In addition, the influence of urea concentration in alkaline electrolytes (typically 0.33–0.5 M) on catalytic performance may be partially related to differences in pore size and pore distribution. Catalysts with hierarchical mesoporous or macroporous structures generally exhibit improved tolerance to higher urea concentrations due to enhanced reactant diffusion and reduced mass‐transport limitations, whereas micropore‐dominated structures may experience diffusion constraints under concentrated reactant conditions. A well‐designed hierarchical porosity system enhances mass transport, preventing local active site saturation while ensuring uniform reactant distribution.[^92^, ^97^, ^98^] Additionally, the inherent layer‐stacking tendency in 2D materials may lead to partial site blockage, reducing accessibility to catalytic centers.^[99]^ Introducing vertically aligned pores mitigates this issue by facilitating electrolyte infiltration and reactant diffusion, thereby maintaining high catalytic activity.^[100]^ Compared to conventional catalysts, porous 2D materials with optimized pore structures exhibit improved utilization of active sites and superior charge transport properties, making them highly effective in UOR applications.[^101^, ^102^, ^103^] Compared to conventional catalysts, porous 2D materials with optimized pore structures exhibit improved utilization of active sites and superior charge transport properties, making them highly effective in UOR applications. Moreover, recent studies suggest that the individual contributions of different pore sizes can be partially distinguished through combined structural and kinetic analyses. Micropores mainly enhance adsorption and intermediate stabilization, mesopores regulate ion diffusion kinetics as reflected by Warburg impedance in EIS measurements, while macropores primarily influence bulk mass transport and limit current behavior. Correlating pore size distribution with diffusion‐related parameters and intrinsic activity metrics (e.g., turnover frequency (TOF)) provides a semi‐quantitative framework for disentangling pore‐dependent transport effects.

### Surface defects

Surface defects play a fundamental role in modulating catalytic activity by influencing adsorption behavior, charge redistribution, and reaction pathway selectivity.[^104^, ^105^, ^106^] Given the high activation energy barrier associated with urea adsorption and C–N bond cleavage, defect engineering provides an effective strategy for optimizing electronic states and lowering the energy barrier.^[107]^ Surface defects primarily fall into three categories: ^(1)^ cation vacancies, such as missing Ni, Co, or Fe sites, which alter the local coordination environment and enhance electron density, thereby improving urea adsorption and activation; ^(2)^ anion vacancies, such as oxygen or sulfur vacancies, which regulate charge distribution and optimize intermediate binding energies,^[108]^ accelerating C–N bond scission; and ^(3)^ edge and step defects, which feature lower coordination numbers and higher local reactivity, facilitating stronger chemical interactions with urea molecules.[^71^, ^109^] Among these, oxygen vacancies have been widely utilized to improve UOR catalysts.^[110]^ Studies have shown that oxygen‐deficient surfaces enhance charge transfer and modify the oxidation‐reduction behavior of metal centers, enabling more reversible valence state transitions that enhance catalytic stability.[^111^, ^112^, ^113^] Additionally, computational studies reveal that oxygen vacancies generate localized electric field effects, promoting efficient urea activation and intermediate decomposition.^[114]^ However, defect stability remains a key challenge, as structural rearrangement or passivation during prolonged operation may lead to catalytic deactivation.[^115^, ^116^, ^117^] Therefore, optimizing defect density while integrating defect engineering with other structural modifications is essential for achieving high‐performance and durable UOR catalysts.[^61^, ^118^, ^119^]

While oxygen vacancies are widely recognized to facilitate charge redistribution and lower the activation barrier for C–N bond cleavage in UOR, establishing a unified quantitative relationship between vacancy density and intrinsic activity (e.g., TOF) remains nontrivial. Within specific oxide systems, moderate increases in oxygen vacancy concentration often enhance catalytic performance by optimizing electronic structure and adsorption energetics, whereas excessive vacancy formation may induce structural distortion or overly strong intermediate binding, leading to a volcano‐type activity dependence. Importantly, previous studies on transition metal oxides have demonstrated that catalytic activity correlates more fundamentally with electronic structure descriptors—such as for example, orbital occupancy and metal–oxygen covalency—rather than defect concentration alone[^120^, ^121^] These findings suggest that vacancy‐induced modulation of electronic descriptors provides a more transferable framework for rationalizing activity trends across different oxide families, including systems such as NiMoO_4_ and LaNiO_3_, thereby offering a more generalizable structure–activity paradigm for UOR catalyst design.

### Electronic configuration modulation

The electronic configuration of a catalyst governs its interaction with urea molecules, influences charge transfer dynamics, and dictates reaction pathway selectivity.[^122^, ^123^, ^124^] Therefore, precise modulation of electronic states is crucial for optimizing the UOR catalytic performance.^[125]^ Although electronic structure modulation, such as tuning of the d‐band center, can effectively regulate the adsorption strength of catalytic intermediates, the relationship between modulation amplitude and catalytic activity does not necessarily follow a simple linear trend. In many electrocatalytic systems, the activity‐descriptor relationship often exhibits volcano‐type behavior,^[126]^ where excessively strong or weak adsorption can both limit reaction kinetics. Consequently, the influence of d‐band center modulation on UOR overpotential may vary across different catalyst families, including sulfides and phosphides, due to their distinct electronic structures and metal–ligand interactions. Common strategies for electronic structure tuning include: (1) band structure engineering, where energy levels are adjusted to optimize electron transfer efficiency between the catalyst and adsorbed species; (2) d‐band center modulation, which tailors the electronic occupancy of metal active sites to balance urea and intermediate adsorption strengths[^127^, ^128^]; and (3) spin‐state engineering, where transition metal centers are tuned to optimize electronic spin states, improving electron donation and enhancing oxidation kinetics.[^129^, ^130^] Both theoretical and experimental studies confirm that optimizing electronic configurations can significantly reduce the activation energy for C–N bond cleavage while improving catalyst durability.^[63]^ For example, the introduction of oxygen vacancies in transition metal‐based catalysts can shift d‐orbital occupancy, increasing electron density at active sites and accelerating urea oxidation.[^131^, ^132^] Additionally, electronic modulation strategies can selectively weaken the adsorption of undesirable intermediates (e.g., HNCO, NH_4_^+^), mitigating catalyst poisoning and extending operational stability.^[133]^ Importantly, electronic structure tuning must be integrated with defect engineering and pore optimization to achieve a synergistic effect, further enhancing the catalytic efficiency and long‐term performance.[^134^, ^135^, ^136^]

### 2.3 Performance benchmarking and structure–activity trends in UOR catalysts

Before establishing a deeper understanding of the structure–activity relationships in porous 2D materials, it is essential to systematically analyze existing performance data to identify trends in catalytic behavior across different material systems.^[84]^ Table 1 summarizes key performance metrics of reported porous 2D materials for UOR, including overpotential, Tafel slope, exchange current density (j_0_), specific current density (j), TOF,^[167]^ Faradaic efficiency, and long‐term stability.^[168]^ A comparative analysis reveals that materials with highly ordered porous architectures tend to exhibit lower overpotentials and enhanced reaction kinetics, indicating that well‐defined porosity improves active site accessibility, facilitates urea diffusion, and accelerates intermediate conversion.[^169^, ^170^] For instance, holey‐NiFe LDH nanosheets and a‐Ni(OH)_2_‐PNF deliver high current densities of 1.48 and 1.477 V at 100 mA cm^−2^, respectively, benefiting from interconnected pore channels that expose abundant active edges. Furthermore, defect engineering plays a crucial role in enhancing catalytic activity.^[171]^ Oxygen‐vacancy‐rich NiMoO_4_ and *β*‐Ni(OH)_2_ nanoflakes exhibit lower Tafel slopes (∼32.5 and 36 mV·dec^−1^), suggesting improved charge transfer dynamics.^[172]^ Additionally, high‐performance catalysts often demonstrate increased exchange current densities and TOF values, reflecting the ability of optimized electronic structures to lower the activation barrier for C–N bond cleavage and accelerate reaction rates.^[173]^ For example, NiFeS_2_/d‐BNNS achieved an impressive urea oxidation current of 189.4 mA·cm^−2^ at only 0.5 A·cm^−2^, highlighting the advantage of synergistic heterointerfaces and defect‐enhanced conductivity. However, long‐term stability assessments indicate that some catalysts with initially high activity suffer from significant performance degradation over extended operation, likely due to structural collapse, surface reconstruction, or active site poisoning.[^174^, ^175^, ^176^] As illustrated by NiMoO_4_ and fullerene quantum dot (FQD)/CoNi‐LDH/NF, both with stabilities below 20 h, the structural integrity under continuous current load remains a bottleneck. Notably, distinct structure–activity trends emerge among different material classes; transition metal oxides and hydroxides generally achieve high catalytic activity at lower potentials, while sulfides and phosphides excel in electronic conductivity and stability.^[177]^ For example, CoS_1.097_/Ni_3_S_2_ and Mn‐Ni_2_P exhibit low Tafel slopes (33.43 and 29.61 mV·dec^−1^, respectively) and high current densities, indicating favorable electronic band structures. These findings further highlight that the catalytic performance in UOR is governed by the interplay among porosity, surface defects, and electronic configurations.^[178]^ Therefore, future catalyst design should focus on precise control over these factors to achieve highly efficient and durable UOR electrocatalysts.^[179]^ However, it should be noted that the majority of reported durability evaluations summarized above are primarily based on ex‐situ electrochemical tests (chronoamperometry/chronopotentiometry and post‐mortem characterization), while systematic in‐situ/operando studies that trace structural evolution in real time remain limited.[^180^, ^181^] Direct evidence for processes such as pore collapse, sulfur leaching, surface reconstruction, or phase transformation under prolonged UOR operation is still scarce for many catalyst systems. Therefore, establishing real‐time structural tracking (e.g., operando XAS/XRD, in situ Raman/FTIR, and electrochemical‐TEM) and standardized post‐mortem analyses is essential to unambiguously identify degradation pathways and to rationalize the pronounced durability differences reported among various catalysts.

**TABLE 1:** Comprehensive overview of electrocatalytic performance of porous 2D materials for electrocatalytic urea oxidation.

Classification	Catalyst	Electrolyte	Potential (V vs. RHE) @ current density	Tafel slope (mV dec^−1^)	Stability@ current density	References
Porous 2D metal oxides and their derivatives	LaNiO_3_ perovskite	1.0 M KOH	1.28 V @ 10 mA cm^−2^	33.1	45 h @ 50 mA cm^−2^	^[137]^
	Nickel‐enriched LaMn_0.2_Ni_0.8_O_3_	1.0 M KOH	1.27 V @ 10 mA cm^−2^	44.6	30 h @ 50 mA cm^−2^	^[138]^
	NiCo_2_O_4_	1.0 M KOH + 0.33 M urea	1.27 V @ 10 mA cm^−2^	88.3	25 h @ 10 mA cm^−2^	^[139]^
	Small‐sized MnO_2_ nanocrystals	1.0 M KOH + 0.5 M urea	1.33 V @ 10 mA cm^−2^	75	20 h @ 10 mA cm^−2^	^[140]^
	Oxygen vacancy‐rich NiMoO_4_	1.0 M KOH + 0.5 M urea	‐	32.5	‐	^[141]^
Porous 2D metal hydroxides	FQD/CoNi‐LDH/NF	1.0 M KOH containing 0.5 M urea	‐	17	15 h @ 15 mA cm^−2^	^[142]^
	Holey NiFe‐LDH nanosheets	1.0 M KOH + 0.33 M urea	1.48 V @ 100 mA cm^−2^	41.7	16 h @ 10 mA cm^−2^	^[143]^
	a‐Ni(OH)_2_ePNF	1.0 M KOH + 0.5 M urea	1.477 V @ 100 mA cm^−2^	30.9	‐	^[144]^
	β‐Ni(OH)_2_ nanoflakes	1.0 M KOH containing 0.33 M urea	‐	36	‐	^[145]^
	Ni(OH)_2_ ‐NMs	1.0 M KOH + 0.33 M urea	1.35 V @ 10 mA cm^−2^	80	‐	^[146]^
Porous 2D metal sulfides	NiFeS_2_/d‐BNNS	1.0 M NaOH containing 0.5 M urea	189.4 mV @ 0.5 A cm^−2^	50.41	900 h @ 10 mA cm^−2^	^[147]^
	Co‐Ni‐S@NF	1.0 M KOH + 0.33 M urea	‐	29.6	100 h @ 100 mA cm^−2^	^[148]^
	CoS_1.097_/Ni_3_S_2_	1.0 M KOH + 0.33 M urea	0.85 V @ 100 mA cm^−2^	33.43	60 h @ 100 mA cm^−2^	^[149]^
	N‐Co_9_S_8_/Ni_3_S_2_/NF	1.0 M KOH + 0.5 M urea	1.40 V @ 10 mA cm^−2^	88.6	20 h @ 20 mA cm^−2^	^[150]^
	(Fe_0.5_Ni_0.5_)_0.96_S/Co_9_S_8_/NF	1.0 M KOH + 0.5 M urea	1.298 V @ 10 mA cm^−2^	28.90	14 h @ 10 mA cm^−2^	^[151]^
	NiS_2_‐MoS_2_	1.0 M KOH + 0.33 M urea	‐	30	‐	^[152]^
Porous 2D metal phosphides	c‐CoNiP_x_/a‐P‐MnO_y_	1.0 M KOH + 0.5 M urea	‐	75	‐	^[153]^
	Ni_2_P–Co_2_P/C	1.0 M KOH + 0.33 M urea	1.27 V @ 10 mA cm^−2^	28.71	200 h @ 100 mA cm^−2^	^[154]^
	Ru‐Co_2_P/N‐C/NF	1.0 M KOH + 0.5 M urea	1.58 V @ 100 mA cm^−2^	65	15 h @ 10 mA cm^−2^	^[155]^
	Mn‐Ni_2_P	1.0 M KOH + 0.33 M urea	1.46 V @ 1000 mA cm^−2^	29.61	10 h @ 100 mA cm^−2^	^[156]^
Porous 2D metal carbides and nitrides	Ni_3_N/Mo_2_N	1.0 M KOH + 0.33 M urea	‐	34.7	40 h @ 120 mA cm^−2^	^[157]^
	Ir‐Ni_3_N	1.0 M KOH + 0.5 M urea	1.37 mV @ 100 mA cm^−2^	27.73	‐	^[158]^
	rGO/Mn‐Ni_3_N	1.0 M KOH + 0.33 M urea	1.305 V @ 10 mA cm^−2^	54.2	50 h @ 10 mA cm^−2^	^[159]^
	WN/Ni_3_C	1.0 M KOH + 0.33 M urea	1.336 mV@ 100 mA cm^−2^	20	20 h @ 10 mA cm^−2^	^[160]^
	Ni/Mo_2_C@CN	1.0 M KOH + 0.5 M urea	1.51 V @ 10 mA cm^−2^	29.2	20 h @ 10 mA cm^−2^	^[161]^
Other emerging porous 2D materials	CoNi‐MOF@CoNi(OH)_2_	1.0 M KOH + 0.5 M urea	1.278 V @ 10 mA cm^−2^	11.2	‐	^[162]^
	NiMn‐MOF	1.0 M KOH + 0.33 M urea	1.459 V @ 1000 mA cm^−2^	19.2	100 h @ 50 mA cm^−2^	^[163]^
	NiPPc/C	1.0 M KOH + 0.33 M urea	1.39 V @ 10 mA cm^−2^	30.4	‐	^[164]^
	a‐NiB_x_	1.0 M KOH + 0.33 M urea	1.4 mV @ 100 mA cm^−2^	22.8	200 h @ 100 mA cm^−2^	^[165]^
	Ce‐CoO NPs/MXene	1.0 M KOH + 0.5 M urea	1.33 V @ 10 mA cm^−2^	80	100 h @ 10 mA cm^−2^	^[166]^

## 3 ADVANCES IN POROUS 2D MATERIALS FOR UREA OXIDATION CATALYSIS

**FIGURE 4:**
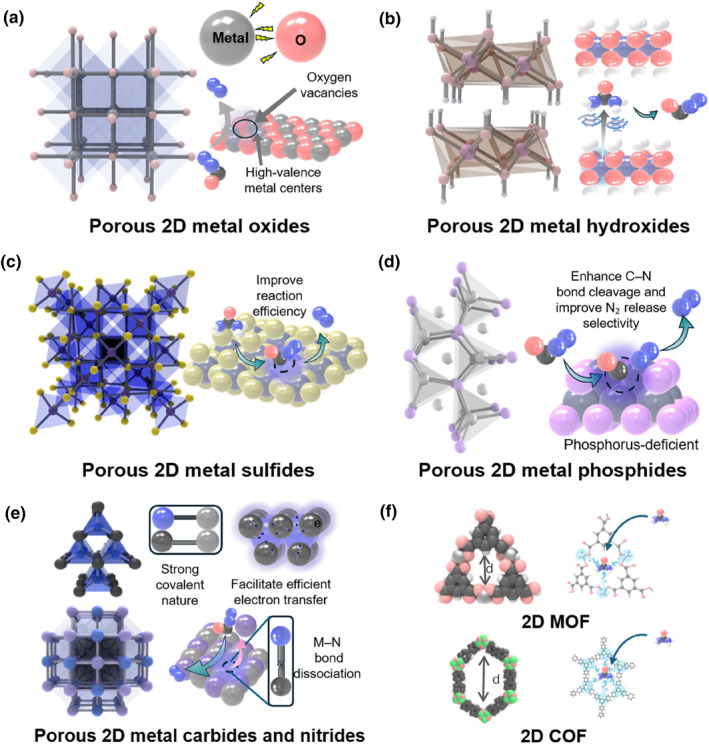
Schematic summary of porous 2D materials for electrocatalytic urea oxidation, classified by material types and structure‐regulation strategies: (a) porous 2D metal oxides, (b) porous 2D metal hydroxides, (c) porous 2D metal sulfides, (d) porous 2D metal phosphides, (e) porous 2D metal carbides and nitrides, (f) porous 2D MOFs and COFs. MOFs, metal–organic frameworks.

Building upon the fundamental understanding of structure–activity relationships in porous 2D materials, it is essential to examine how different material classes contribute to improving the UOR performance. Various porous 2D materials have been explored as electrocatalysts, each exhibiting distinct structural and electronic characteristics that influence catalytic activity, selectivity, and long‐term stability.^[182]^ Figure 4 provides a comparative illustration of the structural features and advantages of different porous 2D material classes, highlighting their unique contributions to UOR catalysis. In this section, we systematically review the latest advancements in six major categories of porous 2D materials: metal oxides and their derivatives, hydroxides, sulfides, phosphides, carbides and nitrides, and other emerging materials. By analyzing their intrinsic properties, catalytic mechanisms, and structure–activity trends, we provide insights into how these materials optimize active site exposure, electronic structure modulation, and durability, ultimately guiding the rational design of high‐performance UOR electrocatalysts.

### 3.1 Porous 2D metal oxides and their derivatives

Porous 2D metal oxides exhibit outstanding electrocatalytic performance for the UOR due to their versatile redox properties, tunable electronic structures, and inherent chemical stability.[^183^, ^184^] Composed primarily of transition metals such as Ni, Co, Fe, Mn, and Mo, these materials benefit from variable oxidation states, allowing them to serve as reversible redox centers that facilitate electron transfer and optimize reaction pathways.^[185]^ Unlike other porous 2D materials, metal oxides possess strong metal–oxygen (M–O) interactions, which play a crucial role in modulating the adsorption strength of urea and its intermediates.[^186^, ^187^] These interactions not only influence catalytic site reactivity but also regulate the electronic environment of active centers during UOR.^[70]^ Additionally, oxygen vacancies and high‐valence metal centers on oxide surfaces contribute significantly to C–N bond cleavage and N_2_ formation.[^111^, ^188^] Oxygen vacancies create localized charge redistribution, optimizing adsorption energies to prevent excessive intermediate accumulation while enhancing charge transport and oxidation kinetics.^[189]^ Moreover, hydroxide‐rich oxide derivatives often form hydrated surface layers under alkaline conditions, improving catalyst durability by mitigating deactivation.[^190^, ^191^] The position of the d‐band center in metal oxides is another key determinant of catalytic activity, as precise tuning of d‐electron occupancy can balance urea adsorption and intermediate conversion,[^192^, ^193^] thereby enhancing selectivity and preventing undesirable byproduct accumulation.^[194]^ Consequently, the unique electronic structure, oxygen vacancy engineering, and stable redox behavior of porous 2D metal oxides and their derivatives make them highly promising candidates for UOR, offering valuable insights for designing efficient and durable electrocatalysts.

**FIGURE 5:**
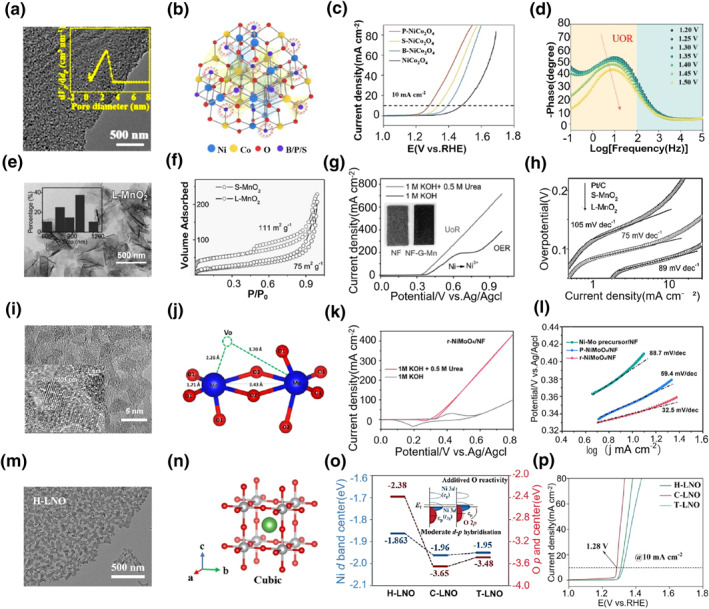
(a) Porous NiCo_2_O_4_ nanosheet morphology. (b) Urea oxidation polarization curves of NiCo_2_O_4_‐based catalysts. (c) Bode phase plots under different applied potentials. (d) Atomic structure model of heteroatom‐modified NiCo_2_O_4_.^[139]^ (Reproduced with permission. Copyright 2023, Elsevier). (e) Large‐sized MnO_2_ nanosheets. (f) Nitrogen adsorption–desorption isotherms of MnO_2_ nanosheets. (g) Urea oxidation activity of Mn‐based electrodes. (h) Tafel plots for urea oxidation on MnO_2_ and Pt/C (Reproduced with permission.^[140]^ Copyright 2016, Wiley‐VCH GmbH). (i) Porous NiMoO_4_ nanosheet morphology.^[141]^ (j) Nitrogen adsorption–desorption isotherms of NiMoO_4_. (k) Urea oxidation polarization curves of r‐NiMoO_4_/NF. (l) Tafel slopes of NiMoO_4_‐based catalysts. (Reproduced with permission. Copyright 2018, American Chemical Society). (m) Hierarchical porous H‐LNO nanosheets. (n) Cubic crystal structure of LNO. (o) Ni 3d and O 2p band center positions of LNO samples. (p) Urea oxidation polarization curves of LNO‐based catalysts.^[137]^ (Reproduced with permission. Copyright 2024, Wiley‐VCH GmbH).

These properties render them highly promising candidates for a range of catalytic processes, thereby prompting further exploration of their practical applications, particularly in the UOR, where recent studies have systematically investigated their efficacy. Wan et al. introduced porous 2D spinel oxides as a model system. As depicted in Figure 5a, high‐resolution TEM images of porous NiCo_2_O_4_ nanosheets reveal a narrow pore‐size distribution, confirming that the microwave shock method effectively facilitates the rapid formation of interconnected mesopores, which promotes electrolyte infiltration and exposes active sites. Additionally, Figure 5b highlights heteroatom‐modified active sites within the spinel lattice, where local coordination distortions at Ni/Co centers optimize the electronic structure for urea adsorption. These structural modifications lead to electrochemical improvements, as evidenced by the reduced onset potential and enhanced anodic current observed in the urea‐containing electrolyte, as shown in Figure 5c. Furthermore, Figure 5d illustrates that the incorporation of heteroatoms in P–NiCo_2_O_4_ simultaneously improves conductivity and intrinsic catalytic activity, without compromising the structural integrity of the porous 2D framework.^[139]^ Building upon the findings of earlier studies, Chen et al. examined the role of lateral size regulation in nanolayers to enhance the UOR activity, demonstrating its significant effect on catalytic performance. Figure 5e presents TEM images of small‐sized (S‐MnO_2_) and large‐sized (L‐MnO_2_) nanolayers, revealing that downsizing results in a substantial increase in edge exposure. This structural modification is further confirmed in Figure 5f, where nitrogen adsorption–desorption isotherms indicate that S‐MnO_2_ exhibits a higher surface area and greater mesoporosity compared to L‐MnO_2_. In terms of catalytic performance, Figure 5g shows a marked anodic response upon the addition of urea, indicating enhanced catalytic activity in S‐MnO_2_. Finally, Figure 5h demonstrates a notable reduction in the Tafel slope for S‐MnO_2_ relative to both L‐MnO_2_ and Pt/C, highlighting that the smaller nanolayers improve mass transport and charge transfer, thereby accelerating the reaction kinetics.^[140]^

In addition to the influence of size effects, defect engineering within porous nanosheets further influences the kinetics of the UOR. Tong et al. synthesized oxygen‐vacancy‐rich r‐NiMoO_4_ porous nanosheets through a controlled reduction process. Figure 5i illustrates the highly porous morphology of these nanosheets, which feature abundant exposed lattice planes. Figure 5j schematically depicts the oxygen vacancies, which generate coordinatively unsaturated metal sites and alter the local electronic environment. These structural modifications result in enhanced catalytic activity, as evidenced by the pronounced urea‐induced anodic response observed in the cyclic voltammetry (CV) measurements shown in Figure 5k. Furthermore, Figure 5l reveals a significant reduction in the Tafel slope compared to both pristine and precursor materials, confirming that the oxygen vacancies facilitate improved charge transfer and accelerate the surface reaction rates.^[141]^ Building upon the strategies of size regulation and defect engineering, Wu et al. introduced crystal phase engineering as a means to precisely modulate the electronic structure of porous 2D perovskites. As shown in Figure 5m, TEM images confirm that various LaNiO_3_ phases retain a 2D porous morphology following microwave shock synthesis. Figure 5n highlights the intrinsic crystallographic differences, with refined single‐cell structures revealing distinct stacking sequences and NiO_6_ octahedral distortions. These structural variations lead to systematic electronic modulation, as demonstrated in Figure 5o, where trends in the Ni d‐band and O p‐band centers suggest that the cubic phase achieves an optimal balance between metal–oxygen covalency and charge‐transfer energy. Consequently, Figure 5p illustrates that cubic LaNiO_3_ exhibits the lowest onset potential and the highest UOR current density among the three phases, underscoring its superior catalytic performance.^[137]^

### 3.2 Porous 2D metal hydroxides

Porous 2D metal hydroxides exhibit unique catalytic properties for the UOR due to their layered structure, high density of hydroxyl (‐OH) groups, and reversible redox activity.[^171^, ^195^] Composed primarily of transition metals, these materials maintain structural stability under alkaline conditions while providing dynamically tunable active sites through redox cycling.^[196]^ Unlike metal oxides, the catalytic performance of metal hydroxides is not solely governed by the redox behavior of metal centers but is also significantly influenced by their layered structure and hydroxyl group interactions.^[197]^ The inherent flexibility of these layers enables adaptive interlayer spacing, which facilitates ion transport and electrolyte penetration, reducing mass transport resistance and enhancing reactant accessibility.^[101]^ Furthermore, surface hydroxyl groups play a key role in stabilizing adsorbed urea molecules through hydrogen bonding networks, thereby lowering the energy barrier for C–N bond cleavage and accelerating reaction kinetics.^[171]^ Mechanistic investigations on Ni‐based hydroxide/oxyhydroxide catalysts have demonstrated that surface hydroxyl species participate in the electrochemical oxidation of urea by facilitating intermediate adsorption and charge‐transfer processes^[198]^ However, these studies primarily focus on elucidating reaction pathways within specific material systems rather than establishing a quantitative relationship between hydroxyl surface coverage and catalytic selectivity (such as suppression of HNCO formation). Comprehensive comparative analyses across different transition‐metal hydroxides are still lacking in the current literature Therefore, although surface hydroxylation is widely considered beneficial for UOR kinetics, whether its influence on selectivity follows a universal trend remains to be systematically clarified. A distinctive advantage of metal hydroxides in UOR is their dynamic redox activity, which allows high‐valence metal species, such as Ni^3+^ and Co^3+^, to actively participate in intermediate transformations and facilitate charge transfer.[^199^, ^200^] Additionally, electrochemical oxidation of hydroxides often leads to the formation of oxyhydroxides (e.g., NiOOH, CoOOH), which exhibit superior hydrophilicity and ionic conductivity compared to metal oxides.[^201^, ^202^, ^203^] These properties enable effective modulation of local electric fields and optimize intermediate adsorption and desorption during UOR.^[204]^ However, the layered structure of metal hydroxides is susceptible to degradation under prolonged electrochemical operation, leading to phase transitions, dissolution, or irreversible structural rearrangements, which compromise long‐term stability.[^40^, ^71^] Strategies such as interlayer modification and surface engineering are therefore essential to enhance durability.^[205]^ Overall, the synergistic interplay of redox tunability, hydroxyl‐mediated adsorption regulation, and enhanced ionic conductivity makes porous 2D metal hydroxides highly promising for UOR catalysis, offering valuable insights for improving activity, selectivity, and stability.^[64]^

**FIGURE 6:**
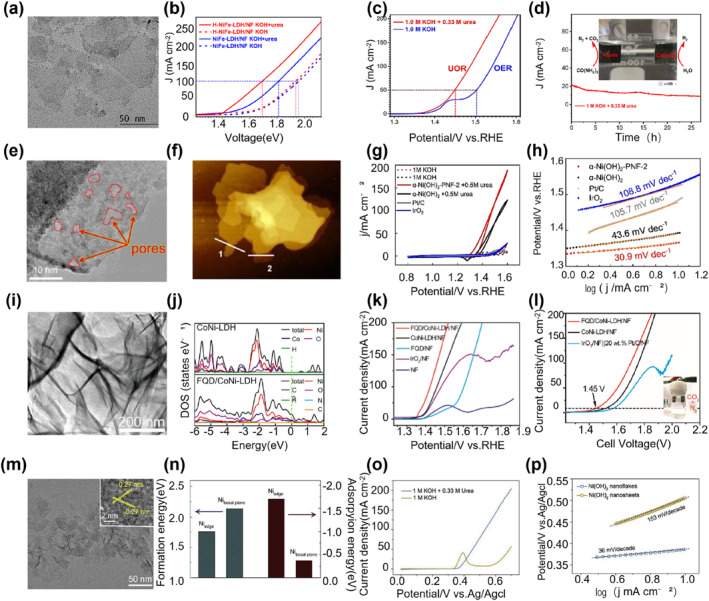
(a) Porous ultrathin NiFe‐LDH nanosheets. (b) Urea oxidation polarization behavior with and without urea. (c) Potential separation between UOR and OER. (d) Long‐term stability during urea electrolysis.^[143]^ (Reproduced with permission. Copyright 2022, Elsevier). (e) Mesoporous and edge‐rich *α*‐Ni(OH)_2_ nanoflakes. (f) Ultrathin morphology and hierarchical assembly. (g) Enhanced urea oxidation activity of *α*‐Ni(OH)_2_‐PNF‐2. (h) Accelerated UOR kinetics from Tafel analysis.^[144]^ (Reproduced with permission. Copyright 2022, Elsevier). (i) Wrinkled CoNi‐LDH nanosheets. (j) Density of states modulation by FQD incorporation. (k) Improved UOR polarization performance. (l) Reduced cell voltage for urea electrolysis.^[142]^ (Reproduced with permission. Copyright 2020, Elsevier). (m) Ultrathin *β*‐Ni(OH)_2_ nanoflakes with exposed edges. (n) Edge versus basal plane energetics from DFT calculations. (o) Anodic behavior with urea addition. (p) Faster reaction kinetics revealed by Tafel slopes.^[145]^ (Reproduced with permission. Copyright 2019, Elsevier). FQD, fullerene quantum dot; OER, oxygen evolution reaction; UOR, urea oxidation reaction.

Building upon the fundamental properties of porous 2D metal hydroxides, recent advancements have focused on structural modifications, such as the introduction of ordered pores, which further enhance both catalytic performance and material stability in the context of the UOR. Chen et al. first demonstrated that the creation of ordered holes in LDH nanosheets effectively enhances both catalytic activity and durability. Figure 6a illustrates that the symmetric H‐NiFe‐LDH/NF||H‐NiFe‐LDH/NF electrolyzer operates at a significantly lower voltage in the presence of urea compared to pure KOH, highlighting the energetic advantage of substituting the OER with UOR. Figure 6b shows that the anodic current for H‐NiFe‐LDH/NF increases upon urea addition, indicating enhanced mass transport and active‐site accessibility facilitated by the holey 2D architecture. Figure 6c further confirms the structural stability of the catalyst, as the current remains stable during long‐term electrolysis. Finally, Figure 6d visually demonstrates the continuous alkaline urea splitting process, reinforcing the catalyst's robust performance in sustained operations.^[143]^ In addition to holey architectures, defect‐rich porous hydroxides further enhance intrinsic reactivity. Li et al. synthesized amorphous a‐Ni(OH)_2_ porous nanoflowers through a sacrificial templating strategy. As shown in Figure 6e, the nanosheets exhibit abundant nanoscale pores, highlighting a high density of defect‐associated active sites. The ultrathin and irregular thickness observed in Figure 6f suggests the increased edge exposure and shortened diffusion pathways, which are advantageous for enhancing catalytic efficiency. These structural features translate into superior UOR performance, as evidenced by Figure 6g, where a‐Ni(OH)_2_‐PNF‐2 demonstrates a significantly stronger urea‐induced anodic response than Pt/C, IrO_2_, and nonporous Ni(OH)_2_. Furthermore, Figure 6h presents a reduced Tafel slope, indicating the accelerated reaction kinetics and confirming the catalyst's enhanced efficiency.^[144]^

To further enhance the‐transfer efficiency, interfacial electronic coupling has been integrated into hydroxide systems. Feng et al. employed FQDs as a modification strategy, anchoring them onto CoNi‐LDH nanosheets grown on Ni foam. As shown in Figure 6i, the CoNi‐LDH nanosheets are ultrathin and wrinkled, with a high density of exposed edges, providing ample active sites for catalysis. Upon decoration with FQDs, Figure 6j demonstrates enhanced electronic states near the Fermi level, suggesting that the interfacial charge transfer between the FQDs and CoNi‐LDH is strengthened. These electronic improvements are reflected in the electrochemical performance depicted in Figure 6k, where the FQD/CoNi‐LDH/NF composite exhibits a higher UOR current density and a lower onset potential compared to the unmodified CoNi‐LDH. Additionally, Figure 6l further validates these enhancements at the device level, showing a reduced cell voltage during urea electrolysis, thereby confirming the effective promotion of charge transfer and overall catalytic performance.^[142]^ Finally, edge‐site engineering provides valuable mechanistic insights into the catalytic activity of hydroxide‐based materials. Yang et al. synthesized ultrathin *β*‐Ni(OH)_2_ nanoflakes through a rapid room‐temperature synthesis method. Figure 6m illustrates the ultrathin platelet morphology, which features a high density of exposed edges, contributing to enhanced catalytic efficiency. Figure 6n presents DFT calculations, which indicate that electroactive NiOOH formation exhibits lower energy at the edges compared to the basal planes, with stronger urea adsorption at the edges. Figure 6o shows a pronounced anodic current induced by urea, further supporting the catalytic benefits of edge sites. Lastly, Figure 6p demonstrates a significantly reduced Tafel slope, providing further evidence that edge‐site engineering promotes accelerated charge transfer and surface reaction kinetics, enhancing the overall catalytic performance of *β*‐Ni(OH)_2_.^[145]^

### 3.3 Porous 2D metal sulfides

Porous 2D metal sulfides exhibit remarkable potential in UOR catalysis due to their high intrinsic conductivity, unique surface chemistry, and tunable electronic structures.[^206^, ^207^, ^208^] Unlike metal oxides and hydroxides, metal sulfides possess lower M–S bond polarity, which enhances electron mobility and facilitates efficient charge transfer at active sites while preventing excessive intermediate stabilization.^[209]^ Their typically narrow bandgaps or metallic‐like electronic structures allow for rapid d‐orbital electron participation, reducing energy barriers for electron transfer and accelerating UOR kinetics.^[210]^ Given that C–N bond cleavage and N_2_ formation involve high activation energy barriers, the enhanced charge transport in metal sulfides significantly improves the reaction efficiency by expediting these key steps.^[133]^ Beyond their superior conductivity, sulfur vacancies play a pivotal role in modulating catalytic activity by altering the local electronic density and shifting the d‐band center, thereby optimizing the urea and intermediate adsorption strengths.[^211^, ^212^, ^213^] This balance prevents excessive intermediate accumulation while promoting efficient electron transfer. Experimental and theoretical studies have demonstrated that sulfur‐deficient catalysts, such as MoS_2_ and Co_3_S_4_, exhibit lower activation barriers for C–N bond scission and enhanced N_2_ release kinetics. Additionally, sulfur vacancies improve the catalyst‐electrolyte interface, increasing wettability and enhancing reactant accessibility, further improving the reaction kinetics. However, metal sulfides are more susceptible to oxidation than oxides or hydroxides, often undergoing structural reorganization or sulfur loss under prolonged electrochemical operation, leading to performance degradation.[^214^, ^215^, ^216^] Strategies such as heterostructure engineering, anion doping, and sulfur vacancy modulation have been explored to enhance long‐term stability.^[217]^ Overall, the combination of high electrical conductivity, defect‐mediated electronic tuning, and optimized charge transfer dynamics makes porous 2D metal sulfides highly effective for UOR catalysis, providing a valuable platform for designing efficient and durable electrocatalysts.

**FIGURE 7:**
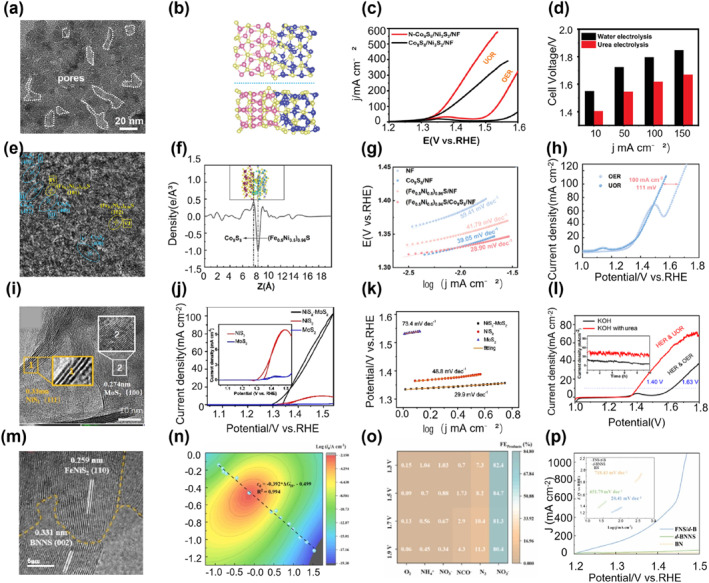
(a) Porous Co–Ni sulfide nanosheets. (b) Interfacial structure of Co_9_S_8_/(Fe_0.5_Ni_0.5_)_0.96_S heterostructure. (c) Urea oxidation polarization curves of Co–Ni sulfide catalysts. (d) Cell voltage comparison of urea electrolysis and water electrolysis.^[150]^ (Reproduced with permission. Copyright 2023, Wiley‐VCH GmbH). (e) Heterointerfacial lattice structure of Co_9_S_8_/(Fe_0.5_Ni_0.5_)_0.96_S. (f) Interfacial charge density redistribution. (g) Tafel slopes of sulfide‐based catalysts. (h) UOR and OER polarization behavior of heterostructured electrodes.^[151]^ (Reproduced with permission. Copyright 2025, Wiley‐VCH GmbH). (i) NiS_2_–MoS_2_ heterostructure with distinct lattice fringes. (j) Urea oxidation activity of NiS_2_–MoS_2_ compared with single components. (k) Tafel analysis of NiS_2_–MoS_2_ catalysts. (l) Two‐electrode performance with and without urea.^[152]^ (Reproduced with permission. Copyright 2021, Elsevier). (m) FeNiS_2_/BNNS heterointerface revealed by HRTEM. (n) Relationship between d‐band center and adsorption energetics. (o) Product FE distribution at different potentials. (p) Urea oxidation performance of BNNS‐coupled sulfide catalysts.^[147]^ (Reproduced with permission. Copyright 2025, Elsevier). FE, Faradaic efficiency; OER, oxygen evolution reaction; UOR, urea oxidation reaction.

Expanding upon the advantageous properties of porous metal sulfides, recent studies have concentrated on advancing their catalytic performance by incorporating sophisticated structural modifications, such as the integration of heterostructures, which further enhance charge transfer dynamics and optimize electrochemical efficiency in the UOR. Xie et al. elucidated the role of porous heterostructures by employing N‐doped Co_9_S_8_/Ni_3_S_2_ nanosheet arrays on Ni foam. As shown in Figure 7a, TEM images reveal a high density of nanoscale pores throughout the nanosheets, confirming that N doping and subsequent post‐treatment result in an open architecture that facilitates enhanced electrolyte penetration and exposes more interfacial sites. Figure 7b provides the electronic rationale behind this enhancement, where structural models indicate that N incorporation induces local coordination perturbations at the Co_9_S_8_/Ni_3_S_2_ interface, thereby promoting charge redistribution and improving urea adsorption. These structural advantages are directly reflected in the electrochemical performance, as shown in Figure 7c, where significantly higher UOR currents are observed at lower potentials compared to OER. Furthermore, Figure 7d demonstrates the enhanced efficiency of urea electrolysis, with substantially lower cell voltages compared to water electrolysis, underscoring the advantages of this engineered heterostructure. Building upon the role of porosity, explicit heterointerface engineering further enhances the kinetics of the UOR.^[150]^ Song et al. synthesized (Fe_0.5_Ni_0.5_)_0.96_S/Co_9_S_8_ heterostructures with intimate phase contact. Figure 7e presents HRTEM and IFFT analyses, which reveal coherent lattice fringes from both sulfide phases, confirming the formation of abundant heterointerfaces. Figure 7f illustrates the differential charge density profiles, showing that interfacial electron transfer is driven by a built‐in electric field, which effectively tunes the adsorption energetics. Figure 7g demonstrates a significant reduction in the Tafel slope for the heterostructures compared to single‐component sulfides, indicating improved electrochemical performance. Furthermore, Figure 7h confirms that UOR proceeds at substantially lower potentials than OER, underscoring the energy‐saving advantages of heterojunction construction.^[151]^

Building upon the progression from interface coupling to phase‐level synergy, Wang et al. engineered NiS_2_–MoS_2_ heterostructures via sulfidation‐induced phase transformation. Figure 7i presents HRTEM images, which reveal the co‐existence of NiS_2_ and MoS_2_ lattice fringes within a single nanostructure, confirming the formation of nanoscale phase coupling. This phase‐level synergy is further evidenced in Figure 7j, where polarization curves show that NiS_2_–MoS_2_ exhibits significantly higher UOR activity than either phase alone. The enhanced catalytic performance is corroborated by the kinetic analysis in Figure 7k, which demonstrates a substantial reduction in the Tafel slope, indicating accelerated charge transfer and enhanced activation of high‐valence Ni species. Finally, Figure 7l shows that urea‐assisted electrolysis effectively lowers the operating voltage for coupled HER–UOR, thereby highlighting the efficiency improvements resulting from phase‐level synergy.^[152]^ Advancing structural regulation to the atomic scale, Hu et al. engineered Fe–Ni sulfide clusters on defective boron nitride nanosheets to precisely modulate adsorption energetics and reaction pathways. Figure 7m displays the well‐resolved lattice fringes of FeNiS_2_ interfaced with BNNS, confirming stable cluster–support coupling. The primary electronic descriptor is elucidated in Figure 7n, where DFT calculations reveal a linear relationship between the d‐band center and adsorption free energy, indicating that the Fe–Ni dual sites facilitate optimized intermediate binding. Further charge density difference analysis reported in the original study shows evident electron redistribution across the Fe–Ni interface, where electrons preferentially transfer from Fe to Ni through bridging S atoms, forming a built‐in interfacial electric field. Bader charge calculations and projected density of states analysis confirm that this intermetallic charge delocalization enhances urea adsorption on the Ni site while facilitating electron back‐donation during C–N bond cleavage, thereby lowering the activation energy barrier.^[218]^ Figure 7o highlights the reaction selectivity, where NO_2_^−^ is the dominant product, with Faradaic efficiencies surpassing 80%. Lastly, Figure 7p shows that the Fe–Ni sulfide clusters exhibit significantly higher UOR current densities and a lower Tafel slope compared to pristine or defect‐only BN, underscoring the enhanced catalytic performance achieved through atomic‐scale structural regulation.^[147]^

### 3.4 Porous 2D metal phosphides

Porous 2D metal phosphides exhibit distinct catalytic advantages in the UOR due to their unique electronic structures, low anionic electronegativity, and excellent chemical stability.^[219]^ Compared to metal oxides and sulfides, metal phosphides possess lower M–P bond polarity, which enhances electron mobility and facilitates a more efficient charge redistribution at catalytic sites.^[220]^ This property reduces the binding strength of reaction intermediates, improving reaction kinetics and catalytic efficiency. Additionally, the lower electronegativity of phosphorus weakens the charge‐shielding effect of metal centers, promoting faster electron transfer and enhancing the intrinsic conductivity of the catalyst. Since UOR involves multiple electron transfer steps, particularly during C–N bond cleavage and intermediate oxidation, the high charge mobility of metal phosphides accelerates the reaction kinetics and stabilizes the redox cycles of metal centers.[^221^, ^222^] The incorporation of phosphorus further modulates the d‐orbital electron distribution, optimizing adsorption energies and facilitating the rapid transformation of urea and its oxidation intermediates, thereby improving the reaction selectivity and catalyst stability.^[223]^ Beyond electronic structure optimization, phosphorus vacancies play a crucial role in modulating catalytic activity by redistributing local charge density and increasing electron‐donating capability.^[224]^ These vacancies adjust the adsorption configurations of urea molecules, reduce activation barriers for key intermediates, and mitigate excessive byproduct accumulation. Studies have demonstrated that phosphorus‐deficient Ni_2_P and FeP catalysts enhance C–N bond cleavage and improve N_2_ release selectivity, thereby significantly boosting UOR efficiency. Furthermore, metal phosphides exhibit superior corrosion resistance compared to oxides and sulfides, maintaining structural stability under both alkaline and neutral conditions, which enables sustained catalytic performance over prolonged cycles.^[225]^ However, surface oxidation of metal phosphides during UOR can lead to the formation of phosphate or hydroxide phases, diminishing catalytic activity. Therefore, stabilizing active sites through phosphorus‐oxygen synergy, vacancy engineering, or heterostructure design is essential for enhancing long‐term performance.[^226^, ^227^] Overall, the high conductivity, tunable phosphorus vacancies, and robust redox properties of porous 2D metal phosphides make them highly promising candidates for UOR, providing valuable insights for designing efficient and durable electrocatalysts.

**FIGURE 8:**
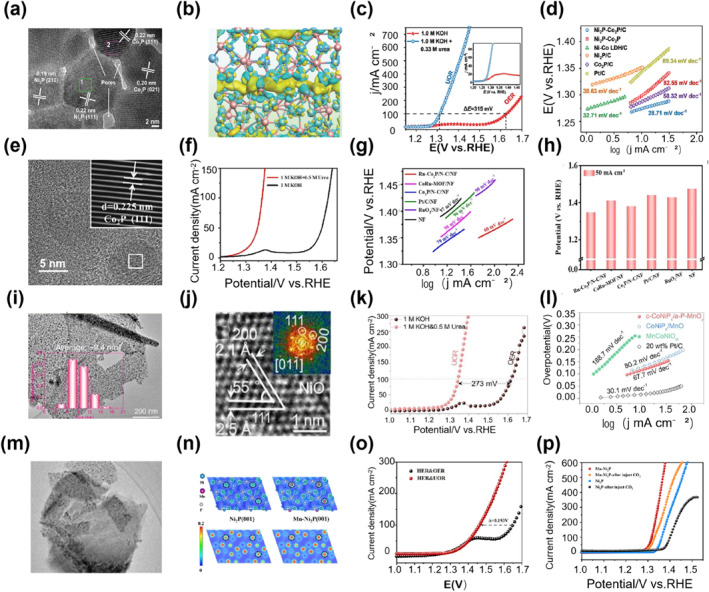
(a) Porous Ni_2_P–Co_2_P nanosheets. (b) Atomic model of the Ni–Co–P heterostructure. (c) Urea oxidation polarization curves. (d) Tafel plots for urea oxidation.^[154]^ (Reproduced with permission. Copyright 2023, Wiley‐VCH GmbH). (e) Lattice‐resolved Co_2_P nanosheets. (f) urea oxidation reaction (UOR) polarization curves in an alkaline electrolyte. (g) Tafel slopes of phosphide catalysts. (h) Operating potentials at fixed current density.^[155]^ (Reproduced with permission. Copyright 2020, Elsevier). (i) Ni_2_P–MoS_2_ heterostructure morphology. (j) UOR activity of Ni_2_P–MoS_2_ catalysts. (k) Tafel analysis of Ni_2_P–MoS_2_. (l) Overall electrolysis performance with urea.^[153]^ (Reproduced with permission. Copyright 2022, Elsevier). (m) FeNiS_2_/BNNS heterointerface. (n) d‐band center and adsorption energetics. (o) Faradaic efficiency (FE) distribution. (p) Urea oxidation polarization curves.^[156]^ (Reproduced with permission. Copyright 2024, Elsevier).

Expanding upon the beneficial properties of porous 2D metal phosphides, recent investigations have underscored the catalytic advantages of integrating intrinsic porosity within 2D oxide nanosheets, thereby optimizing the UOR through enhanced structural accessibility and improved electronic characteristics. In this regard, Wan et al. were the first to demonstrate that incorporating intrinsic porosity within 2D oxide nanosheets offers a fundamental advantage for UOR. As illustrated in Figure 8a, high‐resolution TEM images of porous NiCo_2_O_4_ nanosheets reveal a narrow pore‐size distribution, confirming that the microwave shock method effectively promotes the rapid formation of interconnected mesopores, which facilitate electrolyte penetration and maximize the accessibility of active sites. Beyond morphological advantages, Figure 8b identifies heteroatom‐modulated active sites within the spinel lattice, where local coordination distortions of Ni/Co centers optimize the electronic structure for enhanced urea adsorption. These coupled structural and electronic effects are further corroborated by electrochemical measurements, as shown in Figure 8c, where a reduced onset potential and enhanced anodic current are observed in urea‐containing electrolyte. Additionally, Figure 8d demonstrates that P–NiCo_2_O_4_ achieves improved conductivity and intrinsic catalytic activity without compromising the integrity of the porous 2D framework.^[154]^ Building on the structural modifications discussed previously, Chen et al. further demonstrated that lateral size regulation serves as a crucial extension of porosity engineering, directly impacting the density and accessibility of active sites. As depicted in Figure 8e, TEM images of small‐sized (S‐MnO_2_) and large‐sized (L‐MnO_2_) nanolayers show that downsizing significantly increases edge exposure. This structural difference is further supported by the nitrogen adsorption–desorption isotherms presented in Figure 8f, which reveal that S‐MnO_2_ has a higher surface area and greater mesoporosity than L‐MnO_2_. Figure 8g illustrates the enhanced electrochemical performance of S‐MnO_2_, as evidenced by a pronounced anodic response upon urea addition, demonstrating improved catalytic activity. Moreover, Figure 8h shows a significantly reduced Tafel slope for S‐MnO_2_ compared to both L‐MnO_2_ and Pt/C, indicating that the size‐induced improvements in mass transport and charge transfer effectively accelerate UOR kinetics.^[155]^

In addition to the effects of morphology and size, defect engineering offers an additional degree of control over surface reactivity. Tong et al. fabricated oxygen‐vacancy‐rich r‐NiMoO_4_ porous nanosheets through a controlled reduction process. As shown in Figure 8i, the nanosheets exhibit a highly porous morphology with abundant exposed lattice planes, which is indicative of a structure that enhances catalytic reactivity. Figure 8j provides a schematic representation of oxygen vacancies, which generate coordinatively unsaturated metal sites and modify local electronic states, thereby improving the catalytic performance. This structural modification is reflected in the electrochemical data presented in Figure 8k, where a strong urea‐induced anodic response is observed in CV, indicating improved catalytic activity. Furthermore, Figure 8l shows a significantly reduced Tafel slope compared to both pristine and precursor samples, confirming that the presence of oxygen vacancies facilitates interfacial charge transfer and accelerates surface reaction kinetics.^[153]^ At a higher structural hierarchy, crystal phase engineering facilitates intrinsic electronic modulation, extending beyond the influence of local defects. Wu et al. demonstrated that phase regulation in porous 2D perovskites significantly alters UOR behavior. Figure 8m presents TEM images, confirming that different LaNiO_3_ phases retain a porous 2D morphology following microwave shock synthesis, thereby maintaining comparable structural frameworks. The intrinsic crystallographic differences between the phases are further elucidated in Figure 8n, where refined single‐cell structures reveal distinct stacking sequences and NiO_6_ octahedral distortions. These structural variations lead to systematic electronic modulation, as evidenced in Figure 8o, where trends in the Ni d‐band and O p‐band centers suggest that the cubic phase achieves an optimal balance between metal–oxygen covalency and charge‐transfer energy. Consequently, Figure 8p illustrates that cubic LaNiO_3_ exhibits the lowest onset potential and the highest UOR current density among the three phases, underscoring its superior catalytic performance.^[156]^

### 3.5 Porous 2D metal carbides and nitrides

Porous 2D metal carbides and nitrides exhibit exceptional catalytic potential for the UOR due to their intrinsic metallic conductivity, superior charge transport properties, and remarkable chemical stability.[^228^, ^229^, ^230^] Unlike metal oxides, sulfides, and phosphides, metal carbides (e.g., MXenes, Mo_2_C, WC) and nitrides (e.g., VN, Mo_2_N, TiN) possess low resistivity and metallic electronic structures, significantly reducing charge transport losses and enhancing electron exchange rates at active sites.[^231^, ^232^, ^233^] This facilitates faster oxidation kinetics and lowers the energy barriers associated with UOR.^[234]^ Furthermore, the strong covalent nature of the M–C and M–N bonds imparts exceptional chemical durability, preventing dissolution, structural degradation, and surface oxidation under alkaline conditions and ensuring prolonged catalytic activity.[^235^, ^236^] Their distinctive electronic configuration, characterized by a lower d‐band center position, optimizes the adsorption energies of urea and its oxidation intermediates, leading to enhanced reaction selectivity and kinetic control. C–N bond cleavage, a critical RDS in UOR, is significantly influenced by the electronic structure of metal carbides and nitrides. Carbides, with their metallic conductivity, facilitate efficient electron transfer while enabling precise modulation of intermediate adsorption through d‐band tuning. In contrast, nitrides can undergo partial M–N bond dissociation in alkaline media, exposing high‐electron‐density metal sites that promote C–N bond scission and optimize electron transfer dynamics. Additionally, both material classes readily form interfacial catalytic sites or alkali metal intercalation layers, enhancing urea adsorption and mitigating the accumulation of undesirable byproducts such as HNCO, thereby improving selectivity and long‐term stability. However, despite their advantages, carbides and nitrides remain susceptible to surface oxidation and hydrolysis under prolonged UOR conditions, leading to potential catalyst deactivation.^[237]^ Strategies such as surface modification, heterostructure engineering, and defect modulation are essential for maintaining catalytic stability.[^238^, ^239^] Overall, the high conductivity, robust covalent framework, and tunable electronic structure of porous 2D metal carbides and nitrides establish them as promising electrocatalysts for UOR, providing valuable design insights for enhancing catalytic efficiency and durability.

**FIGURE 9:**
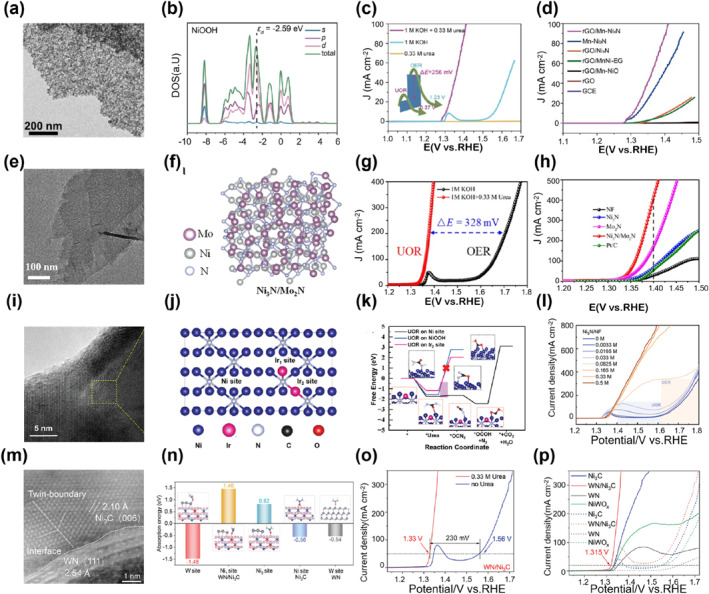
(a) Porous NiOOH‐based nanosheet morphology. (b) Electronic density of states of NiOOH with d‐band center position. (c) Urea oxidation polarization curves in alkaline electrolyte. (d) UOR activity comparison of rGO‐supported catalysts.^[159]^ (Reproduced with permission. Copyright 2023, Elsevier). (e) Layered Ni_3_N/Mo_2_N nanosheet morphology. (f) Atomic model of Ni_3_N/Mo_2_N heterostructure. (g) Polarization curves showing reduced UOR–OER potential gap. (h) UOR activity comparison of nitride‐based catalysts.^[157]^ (Reproduced with permission. Copyright 2023, American Chemical Society). (i) HRTEM image of Ir‐modified Ni_3_N nanosheets. (j) Atomic structure of Ir active sites in Ni_3_N lattice. (k) Reaction energy profiles for urea oxidation on different sites. (l) UOR polarization curves at varied urea concentrations.^[158]^ (Reproduced with permission. Copyright 2024, Wiley‐VCH GmbH). (m) Ni_3_C/WN heterointerface with twin‐boundary structure. (n) Adsorption energies on Ni_3_C/WN heterostructure. (o) UOR polarization curves with and without urea. (p) UOR activity comparison of carbide‐ and nitride‐based catalysts.^[160]^ (Reproduced with permission. Copyright 2023, Wiley‐VCH GmbH). UOR, urea oxidation reaction.

Expanding upon the benefits of porous 2D metal carbides and nitrides, recent researches have concentrated on the development of composite catalysts that improve charge transport and enhance the efficiency of the UOR. Li et al. synthesized rGO‐supported Mn–Ni_3_N catalysts through a nitridation strategy combined with graphene coupling to enhance charge transport and modulate the electronic structure. As shown in Figure 9a, Mn–Ni_3_N nanoparticles are uniformly dispersed on wrinkled rGO sheets, forming a hierarchical structure that maximizes the exposure of active sites. Figure 9b illustrates that Mn incorporation shifts the d‐band center of the reconstructed NiOOH, optimizing the adsorption strength for urea‐derived intermediates. Figure 9c demonstrates that the introduction of urea significantly lowers the anodic potential relative to the OER, confirming the thermodynamic preference of UOR. Lastly, Figure 9d highlights that rGO/Mn–Ni_3_N exhibits higher current densities compared to its undoped counterparts, underscoring the synergistic effects of dopant regulation and conductive carbon coupling.^[159]^ Building upon the concept of interfacial engineering to enhance catalytic performance, Zhang et al. extended this approach to nitride–nitride heterostructures by synthesizing Ni_3_N/Mo_2_N heterojunctions through sequential nitridation, thereby utilizing interfacial electronic coupling. As shown in Figure 9e, Ni_3_N/Mo_2_N retains a lamellar morphology with abundant interfacial contact, which is essential for improving catalytic activity. Figure 9f reveals the atomic configuration of the heterointerface, where charge redistribution facilitates the cooperative adsorption of urea‐related species, optimizing the adsorption dynamics. Figure 9g demonstrates a significant reduction in the operating potential when OER is replaced by UOR, providing evidence of the energetic advantage of heterointerface engineering. Finally, Figure 9h illustrates that Ni_3_N/Mo_2_N outperforms both single‐phase nitrides and Pt/C, underscoring the accelerated reaction kinetics facilitated by efficient interfacial charge transfer.^[157]^

In addition to nitride systems, phase‐coupled hybrids provide an additional dimension for regulating catalytic performance. Wang et al. synthesized crystalline CoNiP nanoparticles confined within an amorphous P‐incorporated MnO matrix through MOF‐assisted phosphorization and controlled oxidation. As shown in Figure 9i, CoNiP nanoparticles are uniformly distributed with an average size of approximately 9.4 nm, indicating effective nanoscale confinement and enhanced accessibility of active sites. Figure 9j illustrates the intimate coupling between the crystalline and amorphous phases, which facilitates interfacial electron exchange. Figure 9k demonstrates a pronounced UOR response at significantly lower potentials than the OER, highlighting the catalytic advantage of the phase‐coupled hybrid structure. Furthermore, Figure 9l shows a reduced Tafel slope compared to single‐component references, confirming that the phase‐coupled hybrid structure accelerates UOR kinetics.^[158]^ At the atomic scale, carbide–nitride interfaces offer an additional mechanism for regulating UOR activity. Liu et al. synthesized WN/Ni_3_C hybrids through a controlled carburization–nitridation process, introducing coherent interfaces and twin boundaries. As shown in Figure 9m, the WN/Ni_3_C interface is well‐defined, with abundant twin boundaries that create structurally distinct active sites. Figure 9n demonstrates that interfacial Ni sites exhibit more favorable adsorption energies for key UOR intermediates compared to isolated metal sites, facilitating enhanced catalytic performance. Figure 9o illustrates that UOR proceeds at significantly lower potentials than the OER, confirming the thermodynamic advantage imparted by interfacial regulation. Finally, Figure 9p shows that WN/Ni_3_C achieves higher current densities than the individual components, highlighting the kinetic benefits of twin‐boundary‐assisted heterointerfaces.^[160]^

### 3.6 Other emerging porous 2D materials

Beyond traditional inorganic systems, a diverse array of emerging porous 2D materials, spanning metal–organic frameworks (MOFs), phthalocyanine nanosheets, and amorphous borides, have demonstrated significant promise in driving the UOR through tailored coordination environments and electronic regulation. Porous 2D MOFs, exemplified by ultrathin CoNi‐MOF and NiMn‐MOF nanosheets, exploit bimetallic synergy and undercoordinated sites to enhance charge transfer and modulate intermediate adsorption. Their open framework and distorted M–O/M–M coordination enabled preferential UOR activation over the OER and reduced Tafel slopes compared to their monometallic counterparts, indicating faster kinetics and improved energy efficiency. The introduction of Mn into Ni‐based MOFs induces local electronic redistribution that optimizes adsorption energies and lowers operating potentials at high current densities, highlighting the effectiveness of heteroatom modulation. In contrast to extended frameworks, molecularly defined 2D phthalocyanine derivatives, such as NiPPc nanosheets, utilize Ni–N_4_ macrocyclic sites embedded within π‐conjugated networks to promote UOR through electron delocalization and orbital stabilization. The planarity and lamellar architecture of these materials ensure maximal exposure of active centers, while the stable Ni–N coordination suppresses overoxidation and maintains catalytic durability. Amorphous porous NiB_x_ films offer a distinct strategy by leveraging short‐range electronic regulation rather than long‐range crystallinity. Boron incorporation modifies the local charge density around Ni centers, stabilizes high‐valence active species, and facilitates C–N bond cleavage while reducing the overpotential and Tafel slope relative to NiO and bare Ni, thus demonstrating a thermodynamic and kinetic advantage in alkaline media.

**FIGURE 10:**
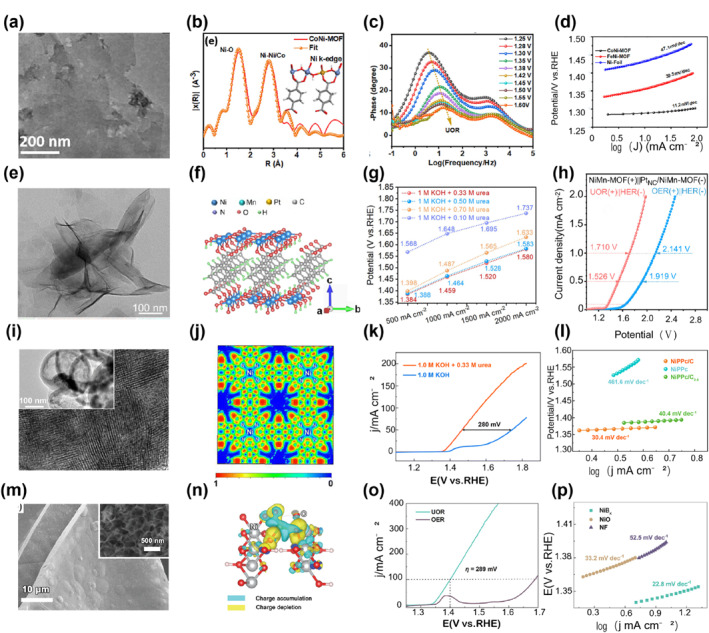
(a) CoNi‐MOF nanosheet morphology. (b) EXAFS spectra of CoNi‐MOF. (c) Bode phase plots for urea oxidation reaction (UOR). (d) UOR Tafel slopes of MOF‐based catalysts. (Reproduced with permission.^[162]^ Copyright 2025, Wiley‐VCH GmbH). (e) Ultrathin NiMn‐MOF nanosheets. (f) Ni/Mn coordination structure. (g) Cell voltages for urea‐assisted electrolysis. (h) Polarization curves for UOR‐coupled electrolysis.^[163]^ (Reproduced with permission. Copyright 2024, American Chemical Society). (i) Porous NiPP/C nanosheets. (j) Charge density distribution of Ni sites. (k) UOR polarization curves. (l) UOR Tafel slopes (Reproduced with permission.^[164]^ Copyright 2025, Royal Society of Chemistry). (m) Porous a‐NiBx nanosheet arrays. (n) Differential charge density around Ni sites. (o) UOR and OER polarization curves. (p) UOR Tafel slopes of a‐NiB_x_.^[165]^ (Reproduced with permission. Copyright 2024, Wiley‐VCH GmbH). OER, oxygen evolution reaction.

Ge et al. synthesized an undercoordinated 2D CoNi‐MOF using a solvent‐regulation strategy, aiming to suppress self‐oxidation and improve UOR performance. As shown in Figure 10a, the CoNi‐MOF exhibits an ultrathin nanosheet morphology, providing a high density of accessible metal sites. Figure 10b reveals the presence of distorted Ni–O and Ni–Ni/Co coordination environments, which confirms the formation of undercoordinated bimetallic sites. Although distorted bimetallic coordination environments are clearly identified, the dominant catalytic motif in emerging 2D MOF/COF systems may still depend on the specific composition and electrochemical conditions. In certain cases, coordinatively unsaturated metal nodes function as the principal adsorption and activation centers, whereas in other systems, conjugated ligands or electrochemically reconstructed M–N/C coordination motifs may participate in the catalytic process. Therefore, definitive assignment of active sites requires element‐ and coordination‐sensitive operando characterization. X‐ray absorption spectroscopy (XANES/EXAFS), for instance, enables direct monitoring of metal valence states and local coordination evolution under working conditions,^[180]^ and when combined with operando vibrational spectroscopy or in situ electron microscopy, can help distinguish intrinsic framework sites from electrochemically transformed species. Density functional theory calculations further indicate that the Co and Ni d orbitals exhibit enhanced overlap near the Fermi level, enabling efficient intermetallic electron delocalization through the MOF coordination network. Differential charge density mapping and XPS binding energy shifts provide experimental and theoretical evidence that electron transfer between Co and Ni centers creates asymmetric charge distribution, which promotes cooperative adsorption of urea by stabilizing the amine group on one metal site and activating the carbonyl group on the adjacent site.^[240]^ Figure 10c demonstrates a distinctive electrochemical response under urea oxidation conditions, indicating a preferential initiation of UOR over the OER. Furthermore, Figure 10d shows that CoNi‐MOF exhibits a smaller Tafel slope compared to both Ni‐MOF and Ni foil, highlighting the accelerated UOR kinetics resulting from its unique structural properties.^[162]^ Building on the concept of bimetallic coordination regulation, Sun et al. synthesized self‐supported NiMn‐MOF nanosheets via solvothermal synthesis to facilitate high‐current‐density UOR. As shown in Figure 10e, the NiMn‐MOF maintained an ultrathin and wrinkled morphology, ensuring abundant exposure of metal nodes. Figure 10f reveals that the incorporation of Mn modifies the local electronic structure of the adjacent Ni centers within the MOF framework, thereby optimizing the catalytic properties. Figure 10g demonstrates that increasing the urea concentration significantly lowers the operating potential over a wide current‐density range, indicating enhanced catalytic efficiency. Moreover, Figure 10h highlights that a NiMn‐MOF‐based electrolyzer achieves a reduced cell voltage for UOR‐coupled electrolysis compared to OER‐based systems, underscoring its improved energy efficiency.^[163]^

Beyond the use of MOF frameworks, precise coordination control at the molecular level further enhances UOR activity. Zhao et al. synthesized planar NiPPc anchored on conductive carbon via surface‐guided molten polymerization. As shown in Figure 10i, the material exhibits a lamellar nanosheet morphology with a well‐defined long‐range order, ensuring maximal exposure of the Ni–N_4_ active centers. Figure 10j reveals significant electron delocalization around the Ni–N coordination environment, indicating that the electronic states are stabilized. Figure 10k demonstrates that the introduction of urea substantially lowers the anodic potential relative to the OER, confirming the preferential activation of UOR. Finally, Figure 10l presents a reduced Tafel slope compared to related references, further validating the enhanced reaction kinetics facilitated using this molecular‐level coordination approach. Amorphous boride systems provide valuable insights into the role of electronic regulation in catalytic performance, independent of long‐range order.^[164]^ Xie et al. synthesized porous amorphous NiBx films using a chemical plating method to modulate the electronic states of Ni. As shown in Figure 10m, the films exhibit an interconnected porous morphology that enhances the electrochemically active surface area. Figure 10n demonstrates the charge redistribution around Ni centers induced by boron incorporation, which stabilizes high‐valence active species. Figure 10o illustrates a significant reduction in overpotential for the UOR compared to the OER, highlighting the thermodynamic advantage of the amorphous boride system. Finally, Figure 10p reveals a smaller Tafel slope for the NiB_x_ films compared to NiO and bare Ni foam, further confirming that electronic modulation facilitates enhanced UOR kinetics.^[165]^

## 4 SUMMARY, CHALLENGES, AND PERSPECTIVES

This review systematically summarizes recent advancements in porous 2D materials for electrocatalytic urea oxidation, emphasizing the influence of structural engineering, electronic modulation, and catalytic mechanisms on performance optimization. Unlike previous studies that primarily focus on material classification or catalytic activity, this work provides an in‐depth analysis of the unique roles of oxides, hydroxides, sulfides, phosphides, carbides, and nitrides in tailoring active sites, optimizing charge transfer, and improving reaction kinetics. Additionally, the interplay between porosity and catalytic behavior is systematically examined, revealing how porous architectures enhance mass transport, increase active site accessibility, and regulate adsorption behavior. By integrating experimental findings and theoretical insights, this review establishes a multi‐scale structure–activity framework to rationalize the effects of microstructural features on catalytic efficiency. Through comparative analysis of different 2D material systems, this review not only advances the fundamental understanding of urea oxidation mechanisms but also provides strategic guidelines for the rational design of high‐performance electrocatalysts.

Despite the promising prospects of porous 2D materials for urea oxidation, several critical challenges remain in catalyst stability, mechanistic understanding, and practical deployment. First, structural evolution, oxidation state transitions, and electronic rearrangements during prolonged operation often lead to instability in catalytic active sites, resulting in performance degradation. Achieving precise control over dynamic active site evolution and designing adaptive or self‐regenerating catalytic materials is essential for long‐term stability. Second, the complex multi‐electron transfer and interfacial adsorption processes in urea oxidation remain insufficiently understood, as most studies rely on empirical optimizations without systematically elucidating electron redistribution, reaction pathway transformations, and intermediate stabilization. Advanced in situ spectroscopic techniques, ultrafast electron microscopy, and computational modeling must be integrated to uncover transient electronic and structural changes during catalysis, providing critical insights for rational catalyst design.^[241]^ Third, while many 2D materials exhibit outstanding catalytic performance in controlled laboratory settings, their scalability, durability, and compatibility with real‐world electrochemical systems remain significant barriers.^[242]^ In particular, the large‐scale fabrication of porous 2D materials remains challenging because many synthesis strategies rely on sacrificial templates, complex multistep procedures, or costly precursors, which increase production costs and limit industrial scalability. In addition, the ultrathin nanosheet structures are prone to restacking or agglomeration during large‐scale synthesis and long‐term operation, which can significantly reduce the accessible surface area and active site exposure. Furthermore, in practical wastewater systems, the presence of impurities such as chloride ions and heavy metal ions may compete with urea adsorption, induce catalyst poisoning, or accelerate structural degradation, thereby affecting catalytic stability and selectivity. Addressing these challenges requires optimizing the structure–interface synergy, developing scalable catalyst fabrication techniques, and integrating modular electrolysis systems for practical applications in wastewater treatment, energy recovery, and sustainable hydrogen production.^[243]^

Future advancements in urea oxidation catalysis will require a paradigm shift in material design and interdisciplinary innovations to achieve breakthrough performance. In catalyst development, constructing dynamically tunable catalysts with environment‐responsive electronic structures will enable self‐adjusting catalytic behavior, ensuring long‐term stability under fluctuating reaction conditions. Additionally, integrating data‐driven strategies such as machine learning‐assisted high‐throughput screening will accelerate the discovery of high‐efficiency catalysts and optimize key structure–activity parameters with unprecedented precision. On a mechanistic level, employing single‐atom catalysis, electric field‐induced pathway modulation, and photo‐electrocatalytic coupling could further lower reaction barriers, enhance charge utilization efficiency, and unlock new reaction pathways. In terms of practical applications, integrating urea oxidation with wastewater valorization, fuel cell technologies, and renewable energy systems could transform the process into a high‐value energy conversion strategy. By leveraging advancements in materials science, computational chemistry, and energy engineering, future research in urea oxidation catalysis is poised to overcome existing limitations and provide sustainable solutions for energy conversion and environmental remediation.

## AUTHOR CONTRIBUTIONS

**Kean Zhu**: Writing—original draft; formal analysis; data curation. **Zebo Li**: Writing—original draft; formal analysis; software. **Ruchuan Chen**: Conceptualization; formal analysis; software. **Linai Zhou**: Formal analysis. **Yujie Ma**: Conceptualization, supervision; validation; writing—review and editing. **Jun Wan**: Conceptualization; funding support; supervision; validation; writing—review and editing.

## CONFLICT OF INTEREST STATEMENT

The authors declare no conflicts of interest.

## Data Availability

Data sharing not applicable to this article as no datasets were generated or analyzed during the current study.
